# Polyurethane membrane with porous surface for controlled drug release in drug eluting stent

**DOI:** 10.1186/2055-7124-18-15

**Published:** 2014-10-08

**Authors:** Eun Ha Seo, Kun Na

**Affiliations:** Department of Biotechnology, The Catholic University of Korea, 43 Jibong-ro, Wonmi-gu, Bucheon-si, Gyeonggi-do, 420-743 South Korea

**Keywords:** Drug eluting stent, Paclitaxel, Polyurethane, Polyethylene glycol, Porous structure, Controlled release

## Abstract

**Background:**

Membrane covered drug eluting stents (DES) were prepared to prevent tumor ingrowth and to control drug release. Polyurethane (PU) is commonly used for DES coating material because of high tensile strength. The release of paclitaxel (PTX) may increase from porous PU membrane.

**Results:**

Polyethylene glycol (PEG) was incorporated into PU membranes to form porous structure and control the release of hydrophobic anti-cancer drug such as PTX. The bare metal stents were coated with PEG incorporated PU and then, PEG was washed out to form porous structure. The crystallization of PTX was inhibited in porous PU membranes and the release of PTX from porous PU membranes was approximately 8.6% more extended over 19 days.

**Conclusions:**

The enhanced release of PTX from porous PU membranes may increase the patency for the DES covering materials.

## Background

Most cancers of extrahepatic bile ducts cause biliary obstruction [1]. The insertion of a bare metal stent is a widely used technique for patients with this malignancy, because this technique prolongs survival, shortens hospital stay, and improves quality of life [2]. However, these stents also have disadvantages of occlusion over time because of tumor ingrowth or overgrowth [3], and mucosal hyperplasia as a consequence of chronic irritation. Moreover, bare metal stents merely promote biliary drainage and have no antitumor effect [4].

Alternatively the local drug delivery system via a stent that is covered with an antitumor-drug-releasing membrane makes it possible to treat a target tissue without adverse systemic effects [5]. The bare metal stent that is covered with paclitaxel (PTX) incorporated membrane that has an antineoplastic effect has been developed [6]. Previously, a polyurethane (PU) membrane was prepared for potential applications to stent-based drug delivery and the local treatment of malignant tumors around non-vascular stents [7]. The PU membrane generally has a high tensile strength that is physically useful as a covering material for gastrointestinal stents that should be compressed inside an introducer tube with a minimum volume during the delivery to the obstructed lumen [8]. Based on the upper reasons, the PU membrane was developed and applied using a dip coating method [9] as part of a PTX-loaded controlled-release membrane for drug-eluting non-vascular stents.

However, the release of PTX was inversely proportional to the PTX loading. This type of the smaller drug release rate with the higher drug loading was reported by S. G. Kang group [10] investigated the percentage of PTX released from PU membrane decreased with the increase in PTX loading. They reported this inverse-relationship of cumulative release % with drug loading is expected, since the amount of drug released from the PU membranes was virtually independent of the drug loading.

Polyethylene glycol (PEG) are commonly incorporated as a pore forming agent to enhance the release of hydrophobic drugs [11]. PEG was incorporated in PU and washed out from PU membranes to form porous structure [12, 13]. The increased surface area of porous structure can facilitate a hydrophobic drug release rate even though higher drug loading [14]. Therefore, we assumed that the porous PU membrane using PEG has possibility to enhance the release of PTX from drug eluting stents (DES).

In this study, the influence of porous structure in the PTX incorporated PU membrane was investigated. Also, the surface morphology and pore size were determined by SEM and drug release behavior was confirmed.

## Methods

### Materials

Polyurethane (PU, Pellethane 2363-80AE, Lubrizol) and bare metal stents were supplied by Teawoong medical co. Ltd. (Kimpo-si, South Korea). Polyethylene glycol (PEG, average Mn 2,050), *tert*-butyl methyl ether (*t*BME), tween 20 was purchased from Sigma Aldrich (St.Louis, MO, USA) and tetrahydrofuran (THF) was purchased from Junsei chemical (Tokyo, Japan). Paclitaxel (PTX) was purchased from Samyang biopharmaceuticals (Seoul, South Korea). All of the other chemicals and solvents were analytical grade.

### Preparation of PU membranes and PU coated bare metal stents

The predetermined amounts of PEG (0, 10, 20, 30%, w/w) and 500 mg of PU (5%, w/v) were dissolved in 10 ml of THF (Table 1). The PU solutions containing various amount of PEG were vigorously stirred to obtain homogenous solution for 24 hours. PU membranes were fabricated using the dip coating technique on a polytetrafluoroethylene (Teflon) bar (Ø: 10 mm). The teflon bars dipped into PU solutions containing PEG of 0, 10, 20 and 30% (w/w) and withdrawn, respectively. The PEG incorporated PU membranes were dried at room temperature for 24 hours [15] and then washed in distilled water for further 24 hours to wash out PEG and form porous structure. The porous PU membrane covered bare metal stents were coated by same methods via coating and washing process of PEG on bare metal stents. Finally, PU membranes and PU membrane covered stents were cut and used for further studies.

**Table 1:** **Composition of PU solution and thickness of PU membranes**

Membrane type (w/w%)	PEG (mg)	PU (mg)	THF (mL)	Membrane thickness (μm) ^a^
PEG 0%	0.0	500.0	10.0	16.0 (±3.6)
PEG 10%	61.1	500.0	10.0	12.7 (±1.2)
PEG 20%	137.5	500.0	10.0	11.3 (±0.1)
PEG 30%	235.7	500.0	10.0	14.0 (±0.1)

^a^Measured by micro-meter caliper.

### Characterization of PU membranes

Thickness of PU membranes was measured by micro-meter caliper (Mitutoyo, Japan). Surface and cross-sectioned morphology of PU membranes and PU coated bare metal stents were observe with field emission-scanning electron microscopy (FE-SEM, Hitachi S-4800, Tokyo, Japan). The membranes and stents were sliced into small pieces (1 cm × 1 cm), mounted on carbon tape, sputter coated with platinum using an ion coater (10 mA, 45 sec), and then observed at an accelerating voltage of 10 kV.

### *In vitro*PTX release test

PTX (50 mg) was added into 10 mL of PU solution containing various amount of PEG. PTX loaded porous PU membrane was fabricated by above-mentioned method. To investigate drug release profile, approximately 0.46 mg/cm^2^ of PTX incorporated porous and non-porous PU membranes were fabricated. The membranes were placed into 15 mL conical tubes, and 10 mL of 0.1% of tween 20 containing 0.01 M phosphate-buffered saline solution (PBST) was added (n = 3). Release test was performed in shaking water bath at 37°C and 50 rpm for 19 days. The PBST in each tube was collected and replaced at specified times. The released PTX was extracted into *t*BME. The *t*BME was completely evaporated at room temperature for overnight and re-dissolved in 200 μL of HPLC grade methanol (Honeywell-Burdick and Jackson). The released PTX was quantified by high performance liquid chromatography (HPLC) equipped with ultraviolet (UV) detector at 227 nm at a flow rate of 1.0 mL/min with HPLC grade methanol as the mobile phase at room temperature [16]. The column was C18 reverse phase column (Thermo Scientific). The HPLC was calibrated with PTX standard solutions of 1 to 100 μg/mL (correlation coefficient *R*^*2*^ = 0.998).

## Results and discussion

### Characterization of PU membranes

**Figure 1:**
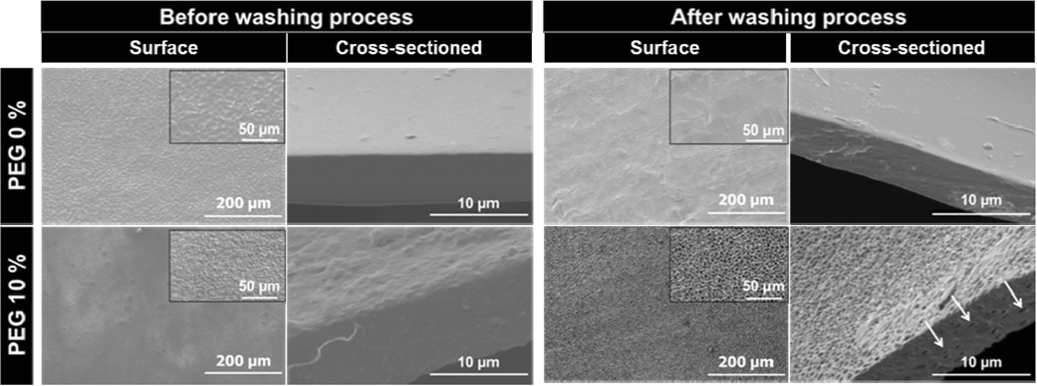
**Surface and cross-sectioned scanning electron micrographs of PU membranes before and after washing process.**

**Figure 2:**
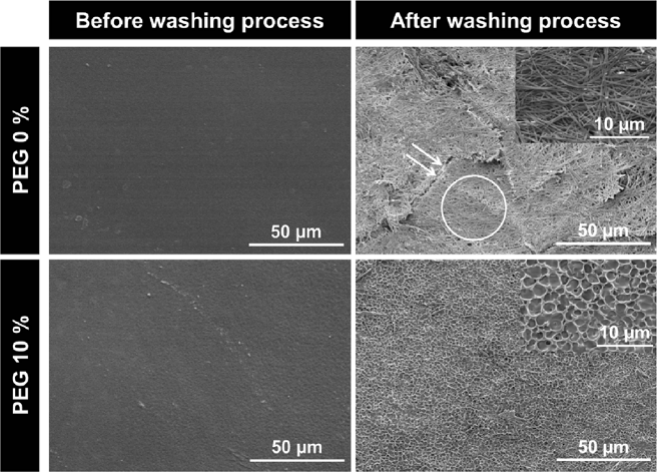
**Scanning electron micrographs of paclitaxel (PTX) incorporated PU membranes before and after washing process.**

The thickness of the PU membranes had a few difference but not significantly affected. PEG 0% PU membrane was 16.0 ± 3.6 μm, PEG 10% was 12.7 ± 1.2 μm, PEG 20% was 11.3 ± 0.1 μm and PEG 30% was 14.0 ± 0.1 μm (Table 1). SEM observation of the PU membranes demonstrated that the surface and cross-sectioned was porous structure after PEG incorporating and washing out from PU membranes (Figure 1). On microscopic examination of the PTX-incorporated PU membranes with SEM, we could find aggregated and cracked PTX for PEG 0% PU membrane, but PTX crystallization wasn’t found on the surface of PEG 10% PU membrane which was a porous membrane (Figure 2). This PTX crystal and cracks allow limited release of PTX from membranes because of rate-limiting detachment of the drug from the PTX-incorporated membranes [17]. In other words, the porous PU membrane can inhibit PTX crystallization and then, PTX release was expected to enhance.

### PU membrane stent cover depending on PEG concentration

**Figure 3:**
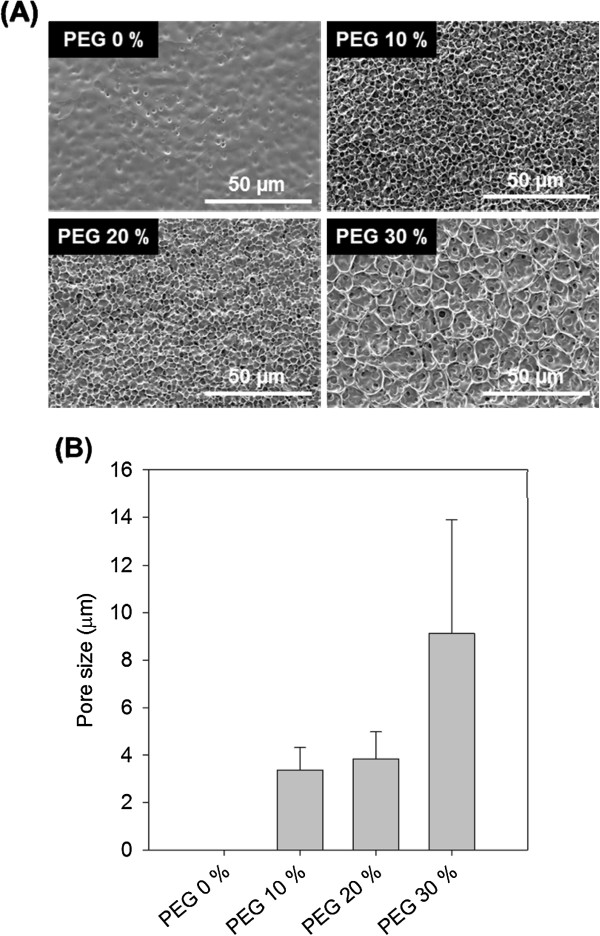
**Determination of pore size of PU membranes after washing process. (A)** Scanning electron micrographs and **(B)** pore size distribution of PU membranes depending on PEG concentration.

**Figure 4:**
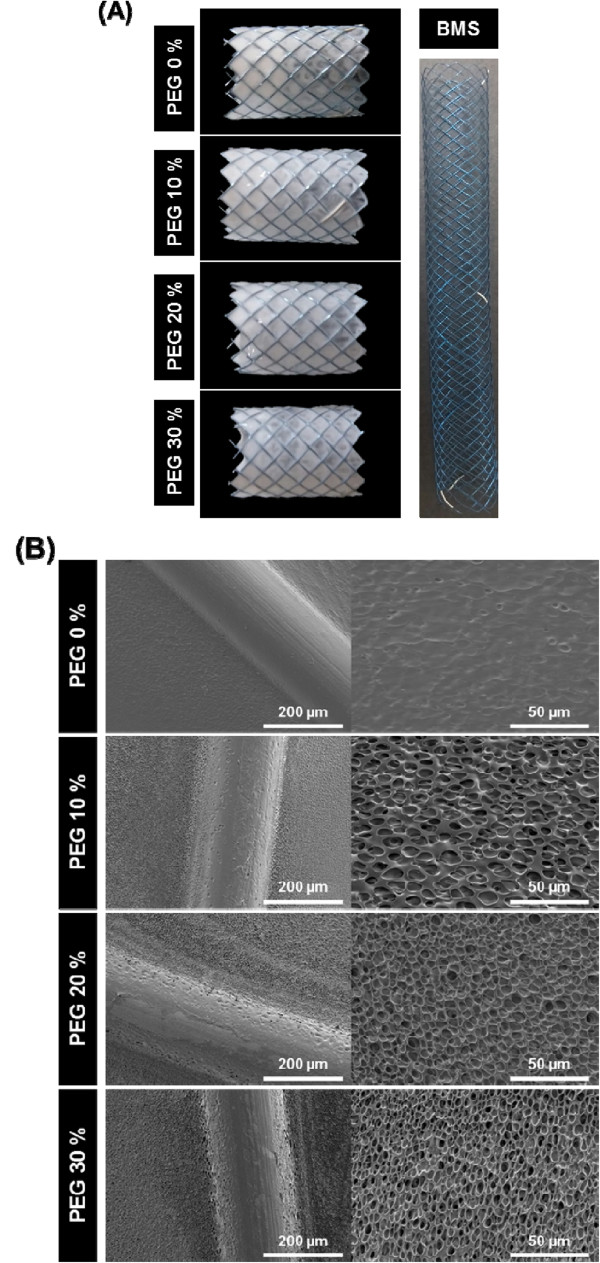
**The porous PU membranes were applied to bare metal stents. (A)** The digital image and **(B)** scanning electron micrographs of PU coated bare metal stents depending on PEG concentration.

Each membrane type has uniform pore size (Figure 3A) and measurement of pore size was increased proportionally to PEG concentration (Figure 3B). PEG was incorporated into PU membrane and washed out in water. The membranes increase in the equilibrium water uptake. This was attributed to the formation of a porous structure in PU membranes. It was also evidenced by the observed increase in the diffusion coefficients. Generally, as diffusion is known to play a major role in the control of drug release [18, 19].According to our stent design, the porous PU membrane was designated as coating membrane on bare metal stent. The PU membrane was formed between metal (Figure 4A). The porous structure of PU membranes coating on bare metal stents can be described like that PU membranes itself, as shown in Figure 4B.

### *In vitro*PTX release test

**Figure 5:**
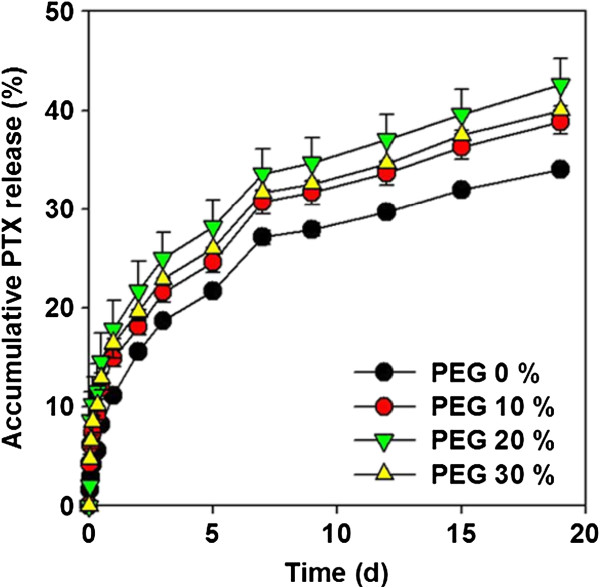
**Accumulative PTX release profile depending on PEG concentration.**

The PTX release behaviors of PU membranes under simulated physiological conditions (PBST, pH 7.4, 37°C) were investigated and compared (Figure 5). Overall PTX loading was approximately 0.461–0.467 mg/cm^2^. PTX loading amounts was 0.465 mg/cm^2^ in PEG 0% PU membrane, 0.467 mg/cm^2^ in PEG 10%, 0.466 mg/cm^2^ in PEG 20% and 0.461 mg/cm^2^ in PEG 30% (Table 2). The released PTX, which was calculated based on the % released for 19 days, was 34.0% (released amount: 0.158 mg/cm^2^) from PEG 0% PU membrane, 38.9% (0.182 mg/cm^2^) from PEG 10%, 42.6% (0.198 mg/cm^2^) from PEG 20%, and 40.0% (0.185 mg/cm^2^) from PEG 30% (Figure 5). PEG 20% membrane showed the greatest release of PTX. Because PEG 30% membranes has too much larger pore size than PEG 10 and 20% membranes so, surface area of PEG 30% membranes is smaller than PEG 20% membranes [20]. As a result of the release properties associated with porous PU membranes, PTX crystallization was protected and increased surface area could more release from PU membranes.

**Table 2:** **PTX-incorporated PU membranes**

Membrane type (w/w%)	PTX amounts (mg/cm ^2^)
PEG 0%	0.465 (±0.002)
PEG 10%	0.467 (±0.002)
PEG 20%	0.466 (±0.001)
PEG 30%	0.461 (±0.009)

## Conclusions

In this study, we investigated the effect of porous PU membrane as a bare metal stent coating material. The porous structure was formed by washing out of PEG from PU membranes. The release of PTX from porous PU membranes was increased for 19 days. This porous PU membrane could inhibit PTX crystallization and increase drug release because porous structure had larger surface area. The enhanced release of drug from porous PU membranes increases the potential usefulness of a bare metal stent cover to limited drug release of hydrophobic anti-cancer drug, PTX.
